# The Importance of Reporting Clinical and Epidemiological Data in Urology: Local Experiences and Insights from the International Literature

**DOI:** 10.3390/medicina56110581

**Published:** 2020-10-30

**Authors:** Márió Gajdács

**Affiliations:** 1 Department of Pharmacodynamics and Biopharmacy, Faculty of Pharmacy, University of Szeged, 6720 Szeged, Hungary; gajdacs.mario@pharm.u-szeged.hu or mariopharma92@gmail.com; Tel.: +36-62-341-330; 2 Institute of Medical Microbiology, Faculty of Medicine, Semmelweis University, 1089 Budapest, Hungary

Pathologies of the genito-urinary tract are responsible for a considerable disease burden worldwide, leading to significant losses of income, lost working days, increased expenditures for national healthcare systems, and decreased quality of life (QoL) in the affected patients [1]. Among these diseases, infections and malignancies in this anatomical region are some of the most important illnesses in human medicine; nevertheless, benign prostate hyperplasia (BPH), erectile dysfunction, hypospadias, urinary incontinence, and vesicoureteral reflux are also relevant disorders affecting millions [2]. Before the 2000s, systematic data collection on the epidemiology of urological ailments was lacking. In 2001, the National Institute of Diabetes and Digestive and Kidney Diseases (NIDDK) initiated the Urologic Diseases in America (UDA), with the aim of quantifying the importance and the burden of urological diseases in the United States, in both medical and financial terms [3]. After the success of the initial project (which was a significant undertaking), the project was renewed in 2007 to further deepen the understanding of the epidemiology and costs of urinary diseases in America. At present, the Global Burden of Disease (GBD) Study provides the most comprehensive data on the disease burden associated with the pathologies of the urinary tract [4]. 

Urinary tract infections (UTIs; see Table 1) are the third most common types of infections worldwide (following infections of the respiratory tract and the gastrointestinal tract), affecting both outpatients (community-acquired UTIs; CA-UTI) and inpatients (hospital-acquired UTIs; HA-UTI) to a considerable degree [5,6]. As a matter of fact, HA-UTIs account for 25–50% of nosocomial infections overall, representing a serious economic and public health issue for healthcare institutions [7]. UTIs are a common reason for visiting physicians and for the prescription of antibiotics, therefore their inappropriate diagnosis may lead to excess consumption of antimicrobial drugs [8,9]. The diagnosis of UTIs is usually based on the clinical presentation and symptoms of the patient, in addition to positive urine analysis result or culture [9]. UTIs should be differentiated as uncomplicated and complicated UTIs, as this classification has important ramifications in therapy; the risk of treatment failure and sequelae is more common in complicated infections [5,6,7,8,9,10]. In essence, all non-uncomplicated UTIs should be considered as complicated infections. During the diagnosis of UTIs, patient factors should also be considered, which may modify the risk of developing these diseases or may affect decision-making processes in diagnostic and therapeutic choices [7,8,9,10,11]. These host factors include underlying conditions, such as the presence of diabetes, immunosuppression or transplantation, spinal cord injuries, malignancies, pregnancy, and the advanced age of the patients. The most common etiological agents in UTIs are well-known; however, there is significant spatio-temporal variation in the distribution of these pathogens and their antibiotic-susceptibility patters [9,12].

As a part of local antimicrobial stewardship-initiatives, Gajdács et al. have aimed to comprehensively characterize the causative agents of CA-UTIs and HA-UTIs in a 1820-bed teaching hospital in Hungary that is responsible for serving tertiary-care for many of the patients in Southern Hungary. At first, the authors have published their results regarding the prevalence and the resistance rates of uropathogenic *E. coli* and *Klebsiella* spp. over a decade-long study period, with an emphasis on the treatment options of extended-spectrum β-lactamase (ESBL)-producing isolates [13,14,15]. *E. coli* isolates were also characterized based on their ability to ferment lactose (to *lac+* and *lac-* isolates, respectively), which is an important biochemical marker of this species [16]. Subsequently, the authors have also assessed the relevance of other Gram-negative bacteria in the same time-frame (2008–2017), i.e., *Citrobacter*-*Enterobacter*-*Serratia* species [17], members of the *Proteae* tribe (*Proteus*-*Providencia*-*Morganella*) [18], and non-fermenting Gram-negative bacteria, as urinary pathogens [19]. With the data collected on resistance rates, Gajdács et al. have performed a secondary analysis of the resistance rates of Gram-negative urinary pathogens, using classical and novel, clinically relevant resistance indicators (usual drug resistance (UDR); difficult-to-treat resistance (DTR); and modified version of DTR (mDTR/mcDTR)) specific to UTIs [20]. Pursuing data to have a complete epidemiological snapshot of the 10-year period, the authors have also reported on the epidemiology of Gram-positive cocci (i.e., staphylococci, streptococci, and enterococci [21]) and *Candida* species as possible pathogens in UTIs [22]. Urine obtained by suprapubic bladder aspiration is an invasive sampling methodology, which allows for sampling without the risk of contamination, and it also allows for the anaerobic processing of the samples. During the analysis of suprapubic urine samples in the respective 10-year period, the authors have also found some anaerobic pathogens (namely, *Finegoldia magna*, *Peptococcus niger*, and *Peptinophilus indolicus*) in upper UTIs [23]. 

Biofilm-formation is an important factor in the initiation and the pathogenesis of catheter-associated UTIs; additionally, biofilms provide protection for these bacteria against the immune cells of the body and the diffusion of antibiotics [9,12]. Behzadi et al. assessed the possible correlation between the production of *n* = 250 mucoid-variant colonies, biofilm-positivity, and the antibiotic-resistant phenotype in uropathogenic *E. coli* isolates [24]. During laboratory assays, 62.0% of isolates produced mucoid colonies and 47.6% of the tested isolates produced biofilm. While there was no association found between biofilm-positivity and resistance to ciprofloxacin, trimethoprim/sulfamethoxazole, and fosfomycin, biofilm-producers were significantly less common among ESBL-positive isolates [24]. The results of this study have been further verified by the findings of Whelan et al. [25]. Prakapaite et al. studied *n* = 100 uropathogenic *E. coli* isolates recovered from uretheral scrapings using phenotypic methods and phylogenetic analyses using multiplex polymerase chain reaction (PCR); they have found elevated resistance-levels to cephalosporins and fluoroquinolones in the UTI-associated serogroups (O15, O22 and O25, respectively), indicating that the use of these drugs as first-line antimicrobials should be re-considered and alternative drugs should be administered in UTIs [26]. Mickymaray and Al Aboody performed an in vitro study to ascertain the antibacterial and anti-oxidant properties of fifteen different spices against relevant uropathogenic bacteria. Out of the tested spices, *Acorus calamus*, *Alpinia galangal*, *Armoracia rusticana*, *Capparis spinosa*, and *Aframomum melegueta* had potent anti-oxidant activities (measured by 2,2-diphenyl-1-picrylhydrazyl (DPPH), 2,20-azinobis-(3-ethylbenzothiazoline-6-sulfonic acid (ABTS) and the total reducing power (TRP) assays), while *A. calamus*, *A. melegueta*, and *C. spinosa* were the most effective as antimicrobials, with potent inhibitory activities against *K. aerogenes*, *P. mirabilis*, and *S. aureus*. They have highlighted that the use of these spices as complementary medicine in UTIs may be worthy of consideration [27]. Kazlauskas et al. determined the value of diuretic ultrasonography in the diagnosis of obstructive hydronephrosis in a cohort of 72 patients (in the study population, hydronephrosis was more commonly unilateral [84.7%, *n* = 61]); as a result of their study, the authors have concluded that diuretic ultrasonography used as an adjunctive investigation method may aid in the prediction of obstructive hydronephrosis [28].

Dženkevičiūtė et al. assessed the association of erectile dysfunction (ED) and asymptomatic cardiovascular illness in Lithuania in a case-control study: carotid intima media thickness and left ventricular mass index were found to be significantly higher in the ED-group, while no differences were seen regarding pulse wave velocity, atherosclerotic plaque count, and other laboratory tests [29]. Their study has concluded that ED (which was associated with a left ventricular mass index) may be a marker of early cardiovascular illness [29]. In the study by Stolic et al., the survival of patients on hemodialysis with ED (an independent risk factor for mortality) was assessed: from the cohort of 70 patients, 60% (*n* = 42) died during the study period (mean age: 57 ± 6.8 years). Overall, their study highlighted that preserved diuresis, lower severity of cardiovascular illness, less-thick intima media in the carotid arteries, and high-quality hemodialysis were all associated with improved survival in patients with hemodialysis [30]. Bener et al. compared the prevalence of ED among men with normal blood pressure and men with hypertension in Qatar, using the International Index of Erectile Function (IIEF) as a scale of sexual dysfunction: among *n* = 296 patients with hypertension, 66.2% had ED, while this ratio was much lower among normotensive patients (*p* < 0.001); additionally, age, highest level of education, presence of T2DM, and current occupation all had significant influence on the level of sexual dysfunction [31]. 

Urologic cancers affect the organs and anatomical structures of the male and female urinary system and the male reproductive organs (see Table 2). The incidence of all urologic cancers is increasing due to the increase in the life expectancy of the population globally [32]. Bladder cancer (where abnormal cell growth is detected in the lining of the urinary bladder) has the highest incidence among urologic malignancies (incidence—360,000, mortality—150,000), while prostate cancer is the second most prevalent male cancer worldwide [32,33]. Bladder cancer is three-to-four times more common in males than in females, usually in patients over 70 years of age [33]. Many of the prostate cancers show slow growth; they do not metastasize, and they do not reduce the quality of life of the affected patients. On the other hand, renal cancers (i.e., renal cell carcinomas; RCC) have the highest mortality rate among urological cancers; renal carcinomas have many histological cell types and may be heterogenous with different biology and therapeutic sensitivities [32,33,34]. In many cases, published data on the incidence and mortality of urologic cancers is hard to interpret and compare, as disease classifications and ever-changing and endpoints used in the respective studies generally do not match [32,33,34].

Padureanu et al. described a case of a 20-year-old patient, with complaints of hematuria, dysuria, and colicky pain, which were later found to be the caused by the urological manifestation of tuberous sclerosis complex (TSC) [35]. Pan et al. assessed the incidence of bladder cancer in patients with Type 2 diabetes mellitus (T2DM) in a study population of 31,932 T2DM patients, compared to the control group of 63,864 patients without diabetes: overall, they have found that T2DM patients do not have a higher risk of developing bladder cancer [36]. Lee et al. studied the potential anticancer properties of epigallocatechin gallate (EGCG) on a human urinary bladder transitional cell carcinoma (TCC) cell line; during the analyses, the expression profiles of mRNAs and microRNAs were analyzed using next-generation sequencing (NGS) and bioinformatics. Overall, they identified several differentially expressed genes in response to EGCG treatment, notably those involved in redox-metabolism, inflammatory response, and nicotinamide adenine dinucleotide (NAD) biogenesis, which may all have important roles in the persistence of malignant cells [37]. Anglickis et al. compared the efficacy of microwave thermal ablation (MWT) with open partial nephrectomy (OPN) in the treatment of small renal tumors patients over 70 years old in the period between January 2012 and January 2019 in Lithuania. In their report, they found that MWT was the more advantageous method overall (assessed by the pain level reported by the patients after surgery (*p* = 0.008), hospitalization, and operation time (*p* < 0.001)), while there were no differences seen in oncological data and the blood loss-estimated glomerular filtration rate [38]. Augliené et al. assessed the survival rate of renal grafts from cadavers in a tertiary-care center in Lithuania over a 7-year period (2007–2013), which included the data analysis of *n* = 186 renal transplant patients. The authors have found nine independent variables associated with worse renal transplant function assessed one year post transplantation, including arteriolar acute graft rejection, an episode of a UTI, advanced age of the donor, advanced age of the recipient, cerebrovascular disease-caused death in the donor, expanded criteria, female gender donors, and hyalinosis [39]. Mansour et al. compared the microbiota composition of cancerous tissues and urine samples collected from bladder carcinoma patients via 16S rRNA gene sequencing, in an attempt to improve screening and monitoring methods of the disease. In their study, the duplicate analysis of the tissues was reproducible, and the results were independent from the tissue collection site. Compared to the urine microbiota, *Akkermansia*, *Bacteroides*, *Clostridium* sensu stricto, *Enterobacter*, and *Klebsiella* were found to be more abundant in tissue samples [40]. Stuopelytė et al. aimed to assess the miRNA expression-profile in the urine and the prostate tissue of prostate cancer patients, with the aim of using it as a non-invasive diagnostic modality. In their study, expression profiles were determined in 13 prostate cancer tissues using microassay analysis, and the highly expressed human miRNAs were selected for additional analysis by PCR. Based on their findings, upregulation of miR-95 was associated with an aggressive clinical presentation of prostate cancer; in addition, increased levels of miR-19a, miR-19b, and miR-21 were also detected in both prostate cancer tissue and the urine of the affected patients. Their results indicated that the measurement of the levels of specific miRNAs may lead to improved detection of prostate cancer [41]. 

The publication of various microbiological and clinical studies in urology from different geographical regions has important ramifications from the standpoint of epidemiology: on the one hand, reported data may influence the development of therapeutic guidelines for UTIs (empiric antibiotic-therapy) and malignancies (including classical cytotoxic drug protocols and next-generation anticancer therapies), both locally and internationally; on the other hand, the relevant stakeholders and government representatives often base their decisions on published evidence [1,5,9,33]. Therefore, novel studies in the field of urology are strongly encouraged to maintain and improve the high standard of patient-care internationally and to ensure continuous information supply for international datasets on the causative agents of UTIs and cancer registries. The registries allow for the accumulation of large datasets; the secondary analysis of these datasets may provide important insights for the future of patient care in this field [1,5,9,33].

## Figures and Tables

**Table 1:** Different types of urinary tract infections.

Asymptomatic bacteriuria
Uncomplicated UTI/cystitis
Recurrent UTIs
Acute/chronic pyelonephritis
Catheter-associated UTIs
Prostatitis
Urosepsis

**Table 2:** Different types of male and female urological cancers.

Bladder cancer
Renal (kidney) cancer
Testicular cancer
Prostate cancer
Penile cancer
Urethral cancer

## References

[1] Schlomer B.J., Copp H.L. (2014). Secondary Data Analysis of Large Data Sets in Urology: Successes and Errors to Avoid. J. Urol..

[2] Okland T., Karimkhani C., Pederson H., Boyers L.N., Rove K.O., Kenny M.C., Steinberg S., Naghavi M., Dellavalle R.P. (2017). Research prioritization of men’s health and urologic diseases. Int. Braz. J. Urol..

[3] Miller D.C., Saigal C.S., Litvin M.S. (2009). The Urologic Diseases in America Project. The demographic burden of urologic diseases in America. Urol. Clin. N. Am..

[4] Ebrahimi H., Amini E., Pishgar F., Moghaddam S.S., Nabavizadeh Y., Aminorroaya A., Fitzmaurice C., Farzadfar F., Nowroozi M.R., Black P.C. (2019). Global, Regional and National Burden of Bladder Cancer, 1990 to 2016: Results from the GBD Study 2016. J. Urol..

[5] Öztürk R., Murt A. (2020). Epidemiology of urological infections: A global burden. World J. Urol..

[6] Flores-Mireles A.L., Walker J.N., Caparon M., Hultgren S.J. (2015). Urinary tract infections: Epidemiology, mechanisms of infection and treatment options. Nat. Rev. Microbiol..

[7] Hooton T.M., Bradley S.F., Cardenas D.D., Colgan R., Geerlings S.E., Rice J.C., Saint S., Schaeffer A.J., Tambayh P.A., Tenke P. (2010). Diagnosis, Prevention, and Treatment of Catheter-Associated Urinary Tract Infection in Adults: 2009 International Clinical Practice Guidelines from the Infectious Diseases Society of America. Clin. Infect. Dis..

[8] Gupta K., Hooton T.M., Naber K.G., Wullt B., Colgan R., Miller L.G., Moran G.J., Nicolle L.E., Raz R., Schaeffer A.J. (2011). International clinical practice guidelines for the treatment of acute uncomplicated cystitis and pyelonephritis in women: A 2010 update by the Infectious Diseases Society of America and the European Society for Microbiology and Infectious Diseases. Clin. Infect. Dis..

[9] Behzadi P., Urbán E., Matuz M., Benkő R., Gajdács M. (2020). The Role of Gram-Negative Bacteria in Urinary Tract Infections: Current Concepts and Therapeutic Options. Adv. Exp. Med. Biol..

[10] Stefaniuk E., Suchocka U., Bosacka K., Hryniewicz W. (2016). Etiology and antibiotic susceptibility of bacterial pathogens responsible for community-acquired urinary tract infections in Poland. Eur. J. Clin. Microbiol. Infect. Dis..

[11] Geerlings S.E. (2016). Clinical presentations and epidemiology of urinary tract infections. Microbiol. Spectr..

[12] Behzadi P., Behzadi E., Yazdanbod H., Aghapour R., Cheshmeh M.A., Omran D.S. (2010). A survey on urinary tract infections associated with the three most common uropathogenic bacteria. Maedica.

[13] Gajdács M., Ábrók M., Lázár A., Burián K. (2019). Comparative Epidemiology and Resistance Trends of Common Urinary Pathogens in a Tertiary-Care Hospital: A 10-Year Surveillance Study. Medicina.

[14] Gajdács M., Ábrók M., Lázár A., Burián K. (2019). Susceptibility patterns of extended-spectrum beta-lactamase-producing (ESBL) urinary pathogens: Single-center experience. Gyógyszerészet.

[15] Gajdács M. (2019). Extra deaths due to pandrug resistant bacteria: A survey of the literature. Egészségfejlesztés.

[16] Gajdács M., Ábrók M., Lázár A., Burián K. (2020). Differential epidemiology and antibiotic resistance of lactose-fermenting and non-fermenting Escherichia coli: Is it just a matter of taste?. Biol. Futura.

[17] Gajdács M., Urbán E. (2019). Resistance Trends and Epidemiology of Citrobacter-Enterobacter-Serratia in Urinary Tract Infections of Inpatients and Outpatients (RECESUTI): A 10-Year Survey. Medicina.

[18] Gajdács M., Urbán E. (2019). Comparative Epidemiology and Resistance Trends of Proteae in Urinary Tract Infections of Inpatients and Outpatients: A 10-Year Retrospective Study. Antibiotics.

[19] Gajdács M., Burián K., Terhes G. (2019). Resistance Levels and Epidemiology of Non-Fermenting Gram-Negative Bacteria in Urinary Tract Infections of Inpatients and Outpatients (RENFUTI): A 10-Year Epidemiological Snapshot. Antibiotics.

[20] Gajdács M., Bátori Z., Ábrók M., Lázár A., Burián K. (2020). Characterization of resistance in Gram-negative urinary isolates using existing and novel indicators of clinical relevance: A 10-year data analysis. Life.

[21] Gajdács M., Ábrók M., Lázár A., Burián K. (2020). Increasing relevance of Gram-positive cocci in urinary tract infections: A 10-year analysis of their prevalence and resistance trends. Sci. Rep..

[22] Gajdács M., Dóczi I., Ábrók M., Lázár A., Burián K. (2019). Epidemiology of candiduria and Candida urinary tract infections in inpatients and outpatients: Results from a 10-year retrospective survey. Cent. Eur. J. Urol..

[23] Gajdács M., Ábrók M., Lázár A., Burián K. (2019). Microbiology of urine samples obtained through suprapubic bladder aspiration: A 10-year epidemiological snapshot. Dev. Health Sci..

[24] Behzadi P., Urbán E., Gajdács M. (2020). Association between Biofilm-Production and Antibiotic Resistance in Uropathogenic Escherichia coli (UPEC): An In Vitro Study. Diseases.

[25] Whelan S., O’Grady M.C., Corcoran D., Finn K., Lucey B. (2020). Uropathogenic Escherichia coli Biofilm-Forming Capabilities are not Predictable from Clinical Details or from Colonial Morphology. Diseases.

[26] Prakapaite R., Saab F., Planciuniene R., Petraitis V., Walsh T.J., Petraitiene R., Semoskaite R., Baneviciene R., Kalediene L., Kavaliasuskas P. (2019). Molecular Characterization of Uropathogenic Escherichia coli Reveals Emergence of Drug Resistant O15, O22 and O25 Serogroups. Medicina.

[27] Mickymaray S., Al Aboody M.S. (2019). In Vitro Antioxidant and Bactericidal Efficacy of Common Spices: Novel Therapeutics for Urinary Tract Infections?. Medicina.

[28] Kazlauskas Y., Cekoulis A., Bilius V., Aglickis M., Verkauskas G. (2019). Diuretic Enhanced Ultrasonography in the Diagnosis of Pyeloureteral Obstruction. Medicina.

[29] Dženkevičiūtė V., Petrulioneiné Z., Saponka V., Kasilevicius V. (2013). Association Between Erectile Dysfunction and Asymptomatic Cardiovascular Damage in Middle-Aged Men. Medicina.

[30] Stolic R.V., Bukumiric Z., Belic B., Odalovic B., Relic G., Sovtic S., Sipic M., Mitrovic V., Krdzic B. (2020). Survival of Patients on Hemodialysis with Erectile Dysfunction. Medicina.

[31] Bener A., Al-Ansari A., Al-Hamaq A.O.O.A., Elbagi I.E.A., Afifi M. (2007). Prevalence of erectile dysfunction among hypertensive and nonhypertensive Qatari men. Medicina.

[32] GBD Chronic Kidney Disease Collaboration (2020). Global, regional, and national burden of chronic kidney disease, 1990–2017: A systematic analysis for the Global Burden of Disease Study 2017. Lancet.

[33] Parkin D.M. (2008). The global burden of urinary bladder cancer. Scand. J. Urol. Nephrol..

[34] Cairns P. (2011). Renal Cell Carcinoma. Cancer Biomark..

[35] Padurenau V., Dragoescu O., Stoenescu V.A., Padureanu R., Pirici I., Cimpenau R.C., Dalia D., Mihailovici A.R., Tomescu P. (2020). Management of a Patient with Tuberous Sclerosis with Urological Clinical Manifestations. Medicina.

[36] Pan Y., Lee C.Y., Lee L.M., Wen Y.C., Huang J.Y., Yang S.F., Hsiao C.H. (2020). Incidence of Bladder Cancer in Type 2 Diabetes Mellitus Patients: A Population-Based Cohort Study. Medicina.

[37] Lee H.Y., Chen Y.J., Chang W.A., Li W.M., Ke H.L., Wu W.J., Kuo P.L. (2019). Effects of Epigallocatechin Gallate (EGCG) on Urinary Bladder Urothelial Carcinoma—Next-Generation Sequencing and Bioinformatics Approaches. Medicina.

[38] Anglickis M., Anglickiené G., Andreikaité G., Skrebunas A. (2019). Microwave Thermal Ablation versus Open Partial Nephrectomy for the Treatment of Small Renal Tumors in Patients Over 70 Years Old. Medicina.

[39] Augliené R., Dalinkaviciené E., Kuzminskis V., Jievaltas M., Peleckaité L., Gryguc A., Stankevicius E., Bumblyté I.A. (2017). Factors influencing renal graft survival: 7-Year experience of a single center. Medicina.

[40] Mansour B., Monyók Á., Makra N., Gajdács M., Vadnay I., Ligeti B., Juhász J., Szabó D., Osztorházi E. (2020). Bladder cancer-related microbiota: Examining differences in urine and tissue samples. Sci. Rep..

[41] Stuopelytė K., Daniunaité K., Jankevicius F., Jarmalaité S. (2016). Detection of miRNAs in urine of prostate cancer patients. Medicina.

